# Detection of AD-specific four repeat tau with deamidated asparagine residue 279-specific fraction purified from 4R tau polyclonal antibody

**DOI:** 10.1007/s00401-019-02012-0

**Published:** 2019-04-20

**Authors:** Momoko Ebashi, Shuta Toru, Ayako Nakamura, Satoshi Kamei, Takanori Yokota, Katsuiku Hirokawa, Toshiki Uchihara

**Affiliations:** 1 Laboratory of Structural Neuropathology, Tokyo Metropolitan Institute of Medical Science, Tokyo, Japan; 2 Department of Neurology, Nihon University School of Medicine, Tokyo, Japan; 3 Department of Neurology, Nitobe Memorial Nakano General Hospital, Tokyo, Japan; 4 Department of Neurology and Neurological Science, Tokyo Medical and Dental University, Tokyo, Japan; 5 Department of Pathology, Nitobe Memorial Nakano General Hospital, Tokyo, Japan; 6 Neuromorphomics Laboratory, Nitobe Memorial Nakano General Hospital, Tokyo, Japan

Tau proteins are classified according to its isoforms, based on the number of tandem repeat (three or four repeat) of the microtubule binding domain [4]. Both three repeat (3R) and four repeat (4R) isoforms are found in Alzheimer disease (AD), while only 4R isoform is found in progressive supranuclear palsy (PSP)/corticobasal degeneration (CBD) and argyrophilic grains [3]. RD4 is a monoclonal antibody that has been raised against the RD4 epitope (VQII*NKKLDLSNVQSKC=*N*-peptide) corresponding to the second microtubule binding domain [2], not found in 3R tau. This translated sequence is modified irreversibly by deamidation of asparagine at N279 to yield aspartic acid at D279 (VQII*DKKLDLSNVQSKC=*d*-peptide), found in AD [5]. Because this deamidation is not found in PSP/CBD [1], specific probing of 4R tau carrying this deamidation (4R-D) will provide a molecular marker for AD [6], distinct from PSP/CBD (Fig. 1). Even though RD4 is highly specific to *N*-peptide without cross reaction to *d*-peptide [2], RD4 positivity is not specific to PSP/CBD brains (Fig. 2b) because non-deamidated 4R tau (4R-N) is also found in AD brains (Fig. 2a) [6, 7]. Curiously, polyclonal antibody raised against *d*-peptide (4R tau polyclonal antibody) immuolabels tau lesions not only in AD brains containing 4R-D and 4R-N (Fig. 2c) but also in PSP brain containing only 4R-N (Fig. 2d) as reported [1]. This apparent cross reaction is possibly explained by the intrinsic nature of this 4R tau polyclonal antibody that may react more or less to both *d*-peptide and *N*-peptide. Interestingly, however, its absorption with *N*-peptide was complete on PSP brains (Fig. 2f), while this absorption was not evident on AD brains (Fig. 2e), suggesting that this 4R tau polyclonal antibody contains subfractions selectively reactive to 4R-N or *d*-peptide.

**Fig. 1:**
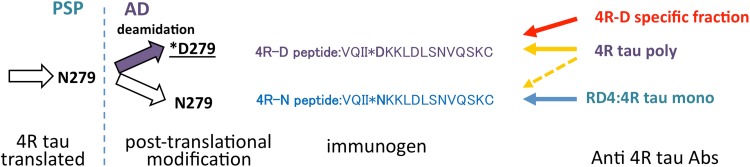
Antigen peptides for 4R tau antibodies

**Fig. 2:**
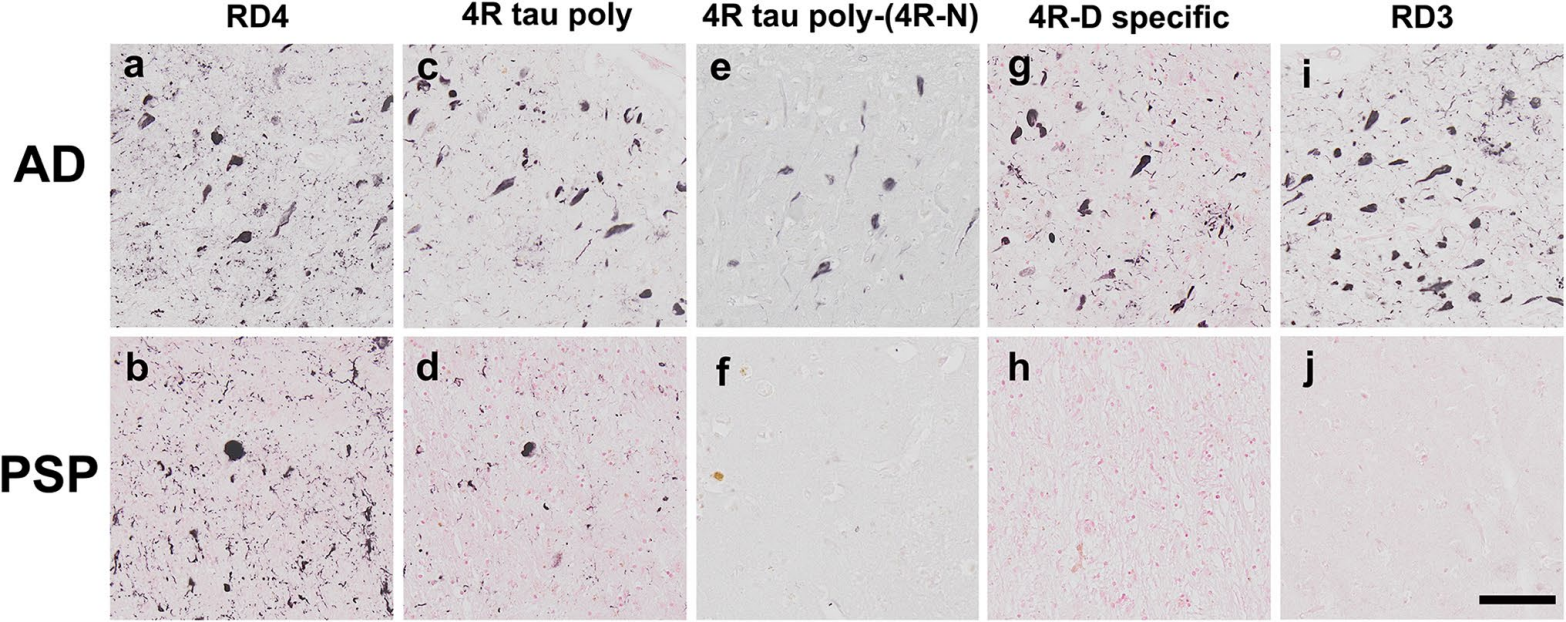
Difference in tau immunoreactivity between AD and PSP brains (bar 100 µm)

Indeed, immunoabsorption of this 4R tau polyclonal antibody with *N*-peptide, followed by affinity purification with *d*-peptide column (Fig. 3) yielded a *d*-peptide specific fraction as confirmed with ELISA (Fig. 4, orange: *d*-peptide; blue: *N*-peptide). As expected, this *d*-peptide specific fraction labeled tau lesions in AD brains (Fig. 2g), but failed to label tau lesions in PSP (Fig. 2h) and CBD brains (data not shown). This contrast in tau immunolabeling, positive with AD brains but negative with PSP brains, is reminiscent of those obtained with RD3 (Fig. 2i, j), while their molecular bases are completely different. This *d*-peptide specific fraction of 4R tau polyclonal antibody clearly confirmed the biochemical contrast that the posttranslational modification at N279D of 4R tau of AD [5] is absent in PSP. Posttranslational deamidation of asparagine into aspartic acid (N279D) may be useful as a biomarker that may distinguish 4R tau of AD from other 4R tau of PSP/CBD. Probing this AD-specific change in 4R tau is a promising way to distinguish AD and PSP/CBD on a molecular basis to be applied for histological, biochemical and biofluid diagnosis (Patent Pending under International Publication number: WO2018/212261).

**Fig. 3:**
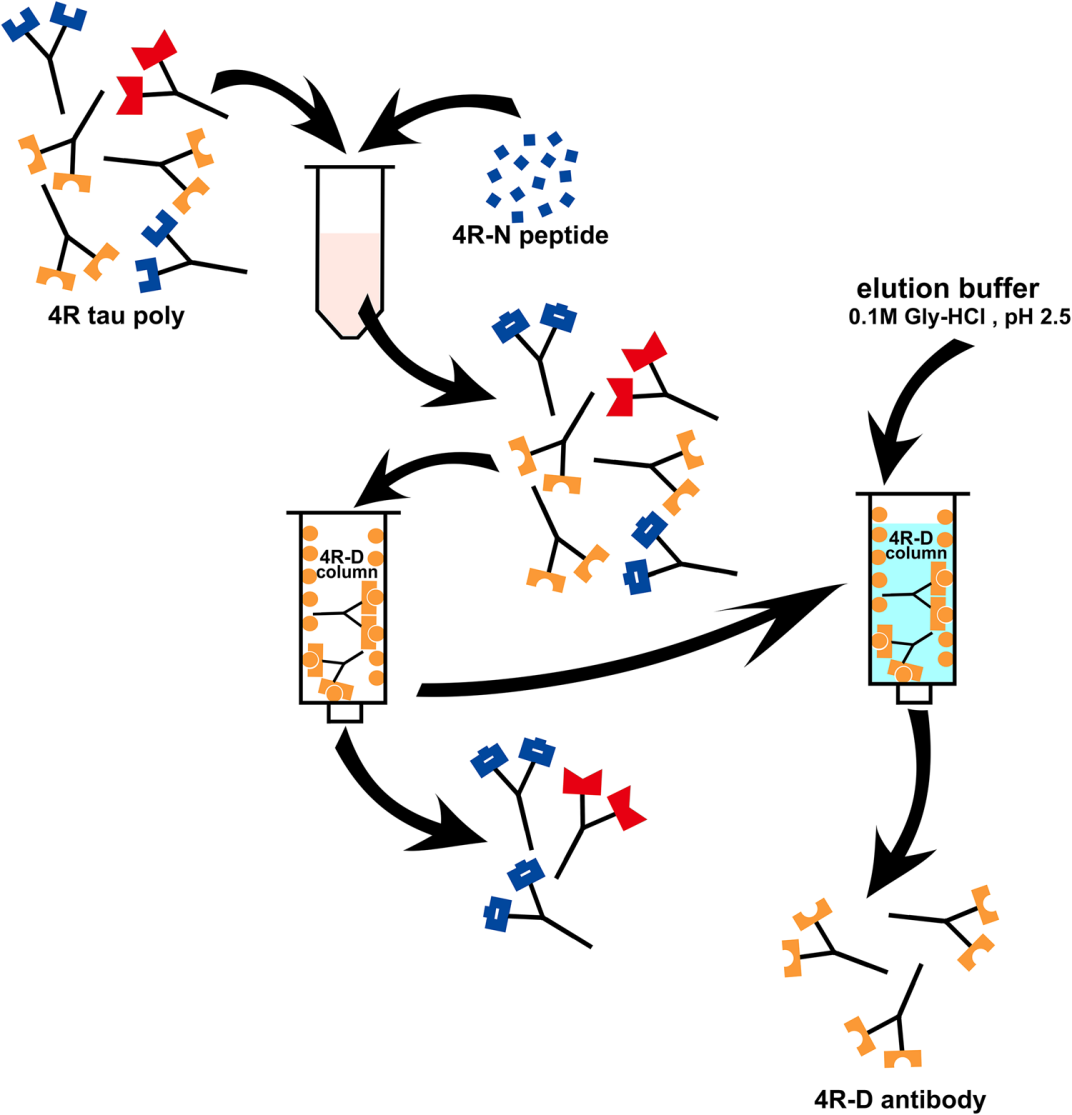
Affinity purification of 4R-D specific fraction from the 4R tau polyclonal antibody

**Fig. 4:**
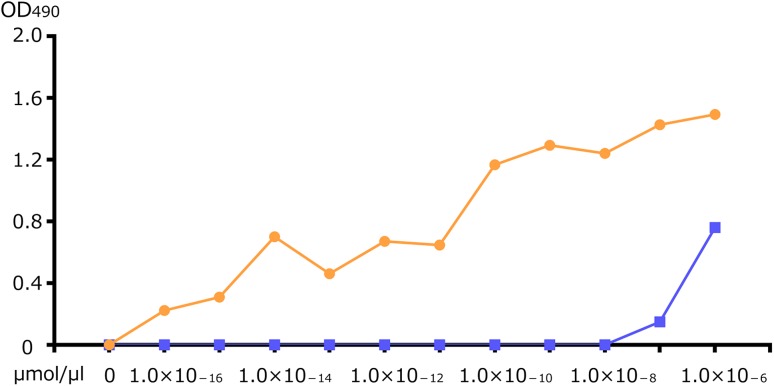
ELISA of the 4R-D specific fraction to 4R-D peptide (orange) and 4R-N peptide (blue)

## Methods

### Peptide synthesis

*N*-peptide corresponding the RD4 epitope (VQII*NKKLDLSNVQSKC) and *d*-peptide, deamidated at N279D (VQII*DKKLDLSNVQSKC), were custom synthesized by Eurofin (Tokyo, Japan, purity > 99%).

### Immunohistochemistry

Formalin-fixed, paraffin-embedded blocks were obtained from four patients of PSP (69–77 yo), a patient of CBD (68 yo), and 4 patients of AD (78–98 yo). Permission of autopsy and of subsequent use for research was obtained from the next to kin of the patients and this study was approved by ethics committee of Tokyo Metropolitan Institute of Medical Science (16-9).

For RD4, serial pretreatment was performed with 0.25% potassium permanganate; KMnO4, for 15 min, 2% oxalic acid for 3 min, > 99% formic acid for 30 min and autoclaved in 0.05 M citrate buffer for 20 min at 120 °C. For 4R tau polyclonal antibody (TIP-4RT-P01, Cosmo bio, Tokyo, Japan) [1], autoclaved in 0.05 M citrate buffer for 10 min at 120 °C and > 99% formic acid for 10 min [8]. Specificity of immunolabeling was confirmed by its disappearance in the presence of the immunogen peptides.

### Affinity purification

After washing formyl cellufine in the column, 200 µg of 4R-D peptide was mixed with 0.6 ml of formyl cellufine in 0.6 ml of coupling buffer (50 mM Na_2_CO_3_·NaHCO_3_, pH 8.5) at room temperature on rotating shaker. After adding 3 mg of sodium cyanoborohydride (NaBH3CN), the formyl cellufine was further shaked at 4 °C for 8–12 h, washed with a blocking buffer (0.1 M monoethanolamine; MEA + 50 mM Tris–HCl buffer, pH 8.0) and mixed with 3 mg of NaBH3CN. This 4R-D peptide primed cellufine was washed with the elution buffer (0.1 M glycine–HCl, pH 2.5) and then with washing buffer (1 M NaCl 1% Triton-X100, pH 7.5) and stored at 4 °C until use. Because 4R tau immunoreactivity on AD brain with the anti-4R tau antibody (1:30,000) was absorbed at the peptide concentration around 1.0 × 10^−7^ µmol/µl, 50 µl of the anti-4R tau antibody was diluted × 8 with 10% bovine serum albumin and mixed with 289.5 µg of 4R-N peptide, equivalent to 1.0 × 10^−7^ µmol/µl at its final concentration. This mixture was reacted with the cellufine primed with 4R-D peptide at 4 °C for 8 h on a rotating shaker. After washing, the bound antibody was eluted with the elution buffer to obtain 4R-D fraction.

### ELISA

The peptide (*N*- or *d*-) was serially diluted to 1.0 × 10^−12~4^ μmol/μl in 50 mM Tris–HCl, pH 8.8 and coated onto microtiter plates (SUMILON) at 4 °C for 16 h. The plates were blocked with 10% fetal bovine serum for 2 h. They were reacted with the purified 4R-D fraction for 90 min at RT followed by incubation with HRP-anti-rabbit IgG made in goat (Bio-Rad) at 1:1000 dilution, and reacted with the substrate, 0.4 mg/ml *o*-phenylenediamine, in citrate buffer (24 mM citric acid, 51 mM Na_2_HPO_4_), The absorbance at 490 nm was measured using Plate Chameleon (HIDEX) as described.
